# Latin-American consensus on the transition into adult life of patients with X-linked hypophosphatemia

**DOI:** 10.1007/s12020-023-03624-z

**Published:** 2023-12-20

**Authors:** Maria Sofia Kastelic, Alejandro Roman-González, Guido De Paula Colares Neto, Francisco J. A. De Paula, Alfredo Adolfo Reza-Albarrán, Lilian Reyes Morales, Silvina Tormo, Adriana Isabel Meza-Martínez

**Affiliations:** 1 Pediatric Endocrinology department, Hospital Nacional Profesor Alejandro Posadas, Buenos Aires, Argentina; 2 Endocrinology department, Hospital Universitario San Vicente Fundación, Medellín, Colombia; 3 Universidad de Antioquia, Medellin, Colombia; 4 Faculdade de Medicina do Centro Universitário São Camilo – SP, São Paulo, Brasil; 5 Department of Internal Medicine, Faculdade de Medicina de Ribeirão Preto-USP, Ribeirão Preto, Brasil; 6 Department of endocrinology and metabolism, Instituto Nacional de Ciencias Médicas y Nutrición Salvador Zubirán, Ciudad de México, México; 7 Chief of the Department of Pediatric Nephrology of the National Institute of Pediatrics, Ciudad de México, México; 8 Department of endocrinology and metabolism, Hospital Nacional Posadas. El Palomar, Buenos Aires, Argentina; 9 Pediatric nephrologist. Instituto Roosevelt, Hospital Infantil Universitario de San José, Bogotá, Colombia

**Keywords:** X-linked hypophosphatemia, Consensus, Transition, Hypophosphatemia, Vitamin D, Bone metabolism disorders

## Abstract

**Introduction:**

X-linked hypophosphatemia is an orphan disease of genetic origin and multisystem involvement. It is characterized by a mutation of the PHEX gene which results in excess FGF23 production, with abnormal renal and intestinal phosphorus metabolism, hypophosphatemia and osteomalacia secondary to chronic renal excretion of phosphate. Clinical manifestations include hypophosphatemic rickets leading to growth abnormalities and osteomalacia, myopathy, bone pain and dental abscesses. The transition of these patients to adult life continues to pose challenges to health systems, medical practitioners, patients and families. For this reason, the aim of this consensus is to provide a set of recommendations to facilitate this process and ensure adequate management and follow-up, as well as the quality of life for patients with X-linked hypophosphatemia as they transition to adult life.

**Materials and Methods:**

Eight Latin American experts on the subject participated in the consensus and two of them were appointed as coordinators. The consensus work was done in accordance with the nominal group technique in 6 phases: (1) question standardization, (2) definition of the maximum number of choices, (3) production of individual solutions or answers, (4) individual question review, (5) analysis and synthesis of the information and (6) synchronic meetings for clarification and voting. An agreement was determined to exist with 80% votes in favor in three voting cycles.

**Results and Discussion:**

Transition to adult life in patients with hypophosphatemia is a complex process that requires a comprehensive approach, taking into consideration medical interventions and associated care, but also the psychosocial components of adult life and the participation of multiple stakeholders to ensure a successful process. The consensus proposes a total of 33 recommendations based on the evidence and the knowledge and experience of the experts. The goal of the recommendations is to optimize the management of these patients during their transition to adulthood, bearing in mind the need for multidisciplinary management, as well as the most relevant medical and psychosocial factors in the region.

## Introduction

Familial X-linked hypophosphatemia (XLH) is an orphan disease of genetic origin, with multisystem involvement, characterized by hypophosphatemia secondary to chronic renal excretion of phosphate, and diminished calcitriol production [1]. X-linked mutations in the phosphate regulating gene with homology to endopeptidases (PHEX) lead to the loss of function of the protein, with increased plasma levels of the fibroblastic growth factor type 23 (FGF23) [1–3]. The excess of FGF23 leads to abnormal phosphate regulation in the kidney, with increased urinary and intestinal phosphorus excretion. Additionally, FGF23 inhibits the 1-hydroxylase enzyme (CYP27B1), hindering vitamin D (1,25 (OH)_2_D) synthesis. This results in a negative effect on phosphorus and calcium absorption in the gut [1, 4].

The incidence of the disease is estimated at 3.9 for every 100,000 live births, with a prevalence ranging between 1.7 and 4.8 for every 100,000 persons (children and adults) [5, 6]. Latin America has few prevalence studies, nevertheless, there is an estimated prevalence of 2.03 for every 100,000 persons in Colombia [7] and of 5 cases per 1,000,000 persons in Brazil [8].

In recent years, as a result of advances in molecular genetics and the diagnosis of these patients, there has been a rise in the prevalence of this disease [5, 9].

Disease manifestations in children usually include *genu varum* or *valgum*, with metaphyseal widening, abnormal cranial morphology secondary to craniosynostosis, impairment of linear growth which conveys a resulting short stature, muscle weakness, bone pain and dental abscesses due to enamel and dentin abnormalities [1, 5, 10–12]. On the other hand, adolescents and adults develop fractures and pseudofractures, with symptoms of osteomalacia and mobility impairment. In adulthood, patients present with progressive hearing loss, osteoarthrosis, enthesopathies, spinal stenosis and motor, neurological and sensory symptoms due to Chiari malformation type 1 and associated syringomyelia [1]. Despite its manifestation in adult life, up until recently, XLH was described as a childhood disease [13].

In recent decades, advances in the management of patients with chronic diseases have resulted in improved survival and quality of life [14–16]. The transition to adult life in patients with XLH (transition defined as the deliberate and planned movement of adolescents or young adults with chronic physical and medical conditions from healthcare systems focused on children to adult-focused care, with or without transfer to a new health service) [17–19] continues to pose a challenge to health systems, practitioners, patients and families because of its multidimensional, multidisciplinary and that requires active family and patient involvement. It is a process that needs to encompass medical, social and educational components of patients and families, focused on care and follow-up during adult life with the aim of providing holistic and comprehensive care and incorporating the transition as part of the care process instituted by the healthcare provider [14, 20].

According to the American Academy of Pediatrics, “The purpose of transition in healthcare for young adults with special health needs is to maximize their lifetime functioning and potential by offering high-quality health services that are adequately developed and continue uninterruptedly while the individual moves from adolescence to the adult stage” [21].

This consensus offers in total of 33 recommendations regarding the transition to adult life in patients with hypophosphatemia, with the aim of optimizing the management of these patients in the Latin-American setting.

## Materials and methods

### Selection of experts

Eight Latin-American experts were asked to participate. They were leaders in the clinical community regarding hypophosphatemia patient management and follow-up. Experts were also chosen to represent geographical regions of Latin America and different medical specialities. Two experts were designated as chairs, and they coordinate the whole process.

### Search strategy

#### This phase comprised two stages: search strategy definition and selection of articles

A systematic search of the literature was conducted in biomedical databases with the aim of identifying clinical practice guidelines, consensus, protocols, algorithms, or expert recommendations on the diagnosis and treatment of hypophosphatemia. The search was circumscribed to publications of the past five years. Embase and Pubmed search engines were used, employing a controlled language depending on the engine (Emtree and MeSH).

#### Selection of articles

Fifty-one papers that met the criteria of approaching the topic of transitioning patients with hypophosphatemia to adult life or that were clinical practices guidelines, consensus, protocols, algorithms or expert recommendations on the topic were identified. The selection was carried out by a single reviewer per reference.

#### Consensus methodology

A version of the Nominal Group Technique (NGT) was used [19–24], structured around four key steps: (1) Individual (silent) generation of ideas, opinions, or answers; (2) review of the answers from the peers, with the opportunity to adjust, modifications or changes to their own individual answers; (3) clarification of opinions and concepts; and (4) voting.

A version supported by information and communication technology (ICT) was implemented, consisting of remote and asynchronic meetings. Remote phases were used for individual production and answer reviews and were carried out through a web application. The clarification and voting phases were synchronic, with the use of teleconferencing and online voting applications that secured anonymity.

A maximum of three voting stages was defined, in each stage, the group of participants used the rating method. In this method, a percentage of rating from 0–100% was assigned to each recommendation by each participant. The rating was average, and an overall score >80% was established as an acceptance of the recommendation by the expert panel. When the average rating was below 30%, the recommendation was eliminated from the next voting stage. If no agreement (50–80%) was reached in the first or second voting round, a process for clarifying arguments was undertaken in order to allow each expert to vote in favor or against during the next stage. In the event of no agreement, after a third voting cycle, the recommendation was stated with the highest number of votes.

An asynchronic vote was taken on SurveyMonkey^®^ between the first and second voting cycles, in order to identify the most favored options among the participating experts.

#### Phases of the consensus

##### Phase 1 – Standardization of the questions

Initial questions were developed regarding each of the necessary considerations for the transition and the transition plan, in accordance with the structure proposed by the “Guideline on the transition between pediatric and adult health care in patients with chronic diseases” [25] and the “Consensus Statement regarding transitions in inborn errors of metabolism” [26], and taking into account the characteristics, barriers, risks and consequences of inadequate transition processes described in the review by Urrea-Sepúlveda and Tovar-Añez [27].

The participants had access to five guidance documents [5, 11, 25–27] for review, apart from the bibliography derived from the review of the literature, and the final questions sent to the experts (Annex A).

##### Phase 2 – Definition of the maximum number of options

A single choice of answer was defined for each of the questions.

##### Phase 3 – Production of individual solutions or answers

During this phase, each expert received the questions together with the instructions to enter their answers or solutions in a web application.

##### Phase 4 – Individual review of the answers

All the participants were able to review the individual answers to each of the questions submitted by the other experts in an anonymous format through the web application. Modifications were then introduced to the individual answers in accordance with this review. Additionally, the participants had the option to review the risk-of-bias graphs of the body of evidence emerging from the review of the articles.

##### Phase 5 – Information analysis and synthesis

The final answers of each of the experts were verified and synthesized by means of affinity matrices. Answers were pooled into single answers according to arrangement and thematic drafting affinity.

##### Phase 6–Synchronic meetings for clarification and voting

The results of phase 5 were presented during this phase. The experts were called to attend three videoconference meetings for voting, clarification and definition of the final recommendations document.

## Recommendations (annex b)

### Recommendation 1

**The following is recommended regarding the minimum level of care or complexity required in institutions that implement plans for transitioning patients with XLH:**

- A long-term care plan which foresees transition to adult outpatient care and requires a level III healthcare service [5, 12, 13, 26–29].
- Adequate multidisciplinary management led by an expert in metabolic bone disorders [5, 13, 26–29].

### Recommendation 2

**The following is recommended regarding the psychosocial factors of patients with XLH which need to be considered to determine the right timing to initiate the transition process:**

- Consider the age of 12 years as the appropriate age to start working with the patient and family on the considerations related to the transition, given that the child is sufficiently ready to understand the process and become actively involved.
- Regarding the assessment of the psychosocial status, the recommendation is to consider the following factors: [13, 25–30]
  ○ The patient’s psychological and emotional maturity.
  ○ The patient’s perception and ability to plan and perform self-care activities.
  ○ The cognitive ability of the patient and caregivers.
  ○ Patient and family ability to acquire and integrate information on the disease and on the indicated medical management and care, and to incorporate it into daily life; the ability to identify problems in the clinical course of the disease and communicate them to the treating physician adequately and on a timely basis.
  ○ Family, personal, school and work support networks and their adequate functioning.
  ○ Cognitive disability or severe psychiatric disorder in the patient or the family/caregiver.
  ○ Sociocultural level of the patient and family.
  ○ The local health system and the capacity to offer an adequate transition of medical care between the pediatric age and adulthood, and the patient’s ability to adapt to this change.

### Recommendation 3

The following clinical criteria are recommended in patients with XLH (disaggregated) in order to determine the right timing for the initiation of the transition process:

- Disease stability: [5, 13, 26, 31].
  ○ Adequate pain control.
  ○ Stabilization of fractures and pseudofractures.
  ○ No need for orthopedic surgery in the immediate future.
  ○ Updated tests and growth curves.
  ○ Stabilization of the calcium-phosphorus metabolism (alkaline phosphatase, parathormone, serum calcium and phosphorus, and calciuria).
  ○ Mobility to allow self-care.
- Treatment stability, with no recent medication or therapy changes [5, 13, 26, 31].
- Absence of complications or clinical deterioration in the past few months [5, 13, 26, 31].

### Recommendation 4

**The following is recommended regarding the average time for the process of transitioning patients with XLH:**

- The length of the transition period should vary depending on individual patient characteristics. It begins in the pediatric age, at around 12 years, when additional information is started to be provided with the aim of promoting autonomy, and ideally extends until 18–21 years of age. (This limit can be extended when needed, regardless of the local administrative criteria) [5, 13, 25, 27, 32].
- The average varies depending on the time when the process is initiated but requires at least 2-3 years and may last up to 6–9 years [25–27, 30, 32].

### Recommendation 5

**The following is recommended regarding the professionals that should be involved in the transition process of patients with XLH:**

- Involve specialists in pediatrics and internal medicine (endocrinology and nephrology) in the process of transitioning patients with XLH to act as leaders of the referring and receiving teams [13, 27, 30].
- Use a targeted and individualized interdisciplinary approach, working with other specialties and professions such as pediatrics, internal medicine, genetics, orthopedics, psychiatry, neurosurgery, otolaryngology, physical medicine and rehabilitation, pain management specialists, psychology, nursing, dentistry, nutrition and dietetics, physical therapy, occupational therapy, ophthalmology, and audiology, depending on individual patient characteristics [5, 26].
- The process should be coordinated with the administrative area of the healthcare institutions involved, through case managers or social workers [11, 26–28].

### Recommendation 6

**It is recommended that at least the following professionals are part of the institutional transition team for patients with XLH:**

- An expert in XLH from the pediatric area (pediatric endocrinologist or nephrologist) and his/her counterpart in adult medicine (endocrinologist or nephrologist) who act as leaders of the “referring and receiving” teams [1, 13, 26, 27].
- A physician, a nurse, a social worker (with experience in transitioning patients with chronic diseases), clinical professionals, volunteers, and administrative staff [1, 13, 26].

### Recommendation 7

**The following is recommended regarding the frequency with which the team must meet to assess and plan actions in the process of transitioning active patients with XLH:**

- It should vary depending on the number of patients who are active in the transition program and their stage in the process.
- Convene the team every 4 months in average. Frequency can vary depending on the local situation in each country.
- Hold one meeting every 3–4 months during the transition period [25, 27].
- Build the transition process during the pediatric stage as part of patient education. It must be intensified starting at 12 years of age to build autonomy conditions for the patient and to enable the pediatric team to prepare appropriately for severing the bond with the patient. Finally, the family should abstain from participating actively in the care of the patient [27].
- Refer the patient from one place to another for future management in hospitals with pediatric and adult services, holding online meetings that can complement face-to-face encounters to make the transition process easier. Online meetings are particularly important in those cases in which transition implies going to a different hospital than the one providing care during the pediatric age [25].

### Recommendation 8

**The following is recommended regarding professionals who participate in the initial assessment of the transition process for patients with XLH:**

- An expert specialized in XLH from the pediatric area, such as a pediatric endocrinologist or nephrologist and his/her adult medicine counterpart such as an endocrinologist or nephrologist. Ideally, they should be accompanied by nursing, social work and mental health professionals [13, 25, 27, 33].
- The person who will act as general coordinator (endocrinologist and/or nephrologist) of the care in the adult hospital, as well as the case manager, should be designated from the start of the process, the use of a care manager arises from the shortage of pediatricians or adult care specialists in some hospital in our region; providing a transversal role in care from the care manager as a key element in countries with heterogeneous health care systems [13, 25].

### Recommendation 9

**The following is recommended regarding the clinical content of the initial assessment carried out as part of the transition process in patients with XLH:**

- A list of clinical data, including potential complications and the established management for the patient [5, 13, 26, 27, 34], as follows:
- Patient identification information.
- Age at the time of diagnosis.
- Complete personal background history (emphasis on the surgical background).
- Complete family background.
- Recent related symptoms.
- Complete physical examination including weight, height, height of the parents, target genetic height.
- Growth curve with growth velocity.
- Result of the genetic test for XLH.
- Laboratory results at the time of diagnosis and of the most recent tests. In serum: phosphorus, calcium, parathormone (PTH), alkaline phosphatase, creatinine, 25 OH vitamin D and 1.25 OH vitamin D. In urine: urine cytochemistry, calcium, phosphorus, creatinine (in isolated urine sample).
- Imaging report: radiographs, renal and urinary tract ultrasound, brain CT or MRI (if available).
- Assessments by other specialists: dentistry, orthopedics, neurosurgery, audiology, with updated opinions.
- Functional tests: Six-minute walk test, Promis (platform to assess health status and the results of the interventions), if available and/or if it has been performed.
- XLH-related complications: Fractures, pseudofractures, lower limb deformities, orthopedic surgeries, dental involvement, auditory impairment, emotional sequelae, and disease burden.
- Treatments received: Time of use, adherence, clinical response and related complications (nephrocalcinosis and/or hyperparathyroidism).
- Surgical treatments received and response to those treatments.

### Recommendation 10

**The following is recommended regarding the psychosocial content of the initial assessment in the process of transitioning patients with X-linked hypophosphatemia:**

- Basic family history: Family structure, roles and basic functioning [13, 27, 34].
- Diagnosis of cognitive impairments or psychiatric diseases in the patient or family members/caregivers [13, 27, 35].
- Adherence to management and follow-up. In case they are inadequate, the reasons should be identified [13, 26, 27, 35].
- Knowledge the patient has regarding the disease, its genetic origin, its chronicity, and the benefit of the treatment.
- The patient’s emotional and psychological maturity: Patient autonomy and ability to provide self-care; education level [6, 13, 27, 35, 36].
- History of support networks for the patient: Presence and contact between the patient and family with XLH groups or organizations (such as support programs for patients with orphan diseases) [13, 27, 35].
- Patient and family opinion regarding the initiation of the transition plan, including expectations, barriers and doubts, among other things [13, 26, 27, 35].

### Recommendation 11

**During the initial assessment in the transition process, it is recommended to evaluate the following aspects related to the therapeutic regimen currently received by the patient with XLH:**

- Individualized treatments received: Calcitriol, phosphate salts or monoclonal antibodies [12, 13].
- Initiation date.
- Dosing.
- Time of use.
- Adverse effects and related complications.
- Adherence to treatment. If inadequate, identify the reasons.
- Assessment of potential treatment changes (in an attempt to improve adherence) or the need to initiate treatment, in case it was not initiated. In the event of treatment change, state the reason.
- Patient and family perception regarding the treatment and its efficacy
- Evaluation by means of quality measures (KPIs) in accordance with pharmacovigilance indicators. For example: Lack of therapeutic efficacy, complications associated with the therapeutic regimen, drug-related allergies and adherence to treatment [33, 37].
- It is important to determine who is responsible for administering the medication (the patient or the guardian) and assess adherence to treatment based on clinical symptoms and laboratory tests (alkaline phosphatase and serum phosphorus), as well as patient knowledge regarding the pharmacological treatment (name, dose, schedule, form of administration, side effects) [12, 13].

### Recommendation 12

**It is recommended to consider genetic counseling for patients with XLH**.

- According to Dahir (2022), genetic counseling must always be offered to patients with X-linked hypophosphatemia at the time of diagnosis [5, 13, 25, 26, 38, 39].
- Genetic assessment must be requested during the transition process (if not done previously) in case of diagnostic doubt, to provide counseling and also expand tests to family members, or for counseling the patient regarding disease transmission (families planning pregnancies or pregnant patients) [5, 13, 25, 26, 38, 39].

### Recommendation 13

**It is recommended to ask for molecular tests in patients with XLH:**

In all cases, if not done during the pediatric age. The team in charge of the transition, attended by the professionals who will continue management during adult life, should be responsible for performing the genetic study [5, 13]. It should be noted, that genetic testing availability can differ between countries, and thus the recommendation may not be fulfilled when the patient transitions; still preforming genetic testing should be done in the future.

***Note***: The implementation of this recommendation may be subject to the guidelines, management protocols, consensus and health system characteristics and regulations in each individual country. Specifically, in Argentina, having a molecular genetic test is not a prerequisite to initiating treatment.

### Recommendation 14

**The following is recommended regarding the role of the transition manager (or case manager) in a transition program for patients with XLH:**

**Fig. 1:**
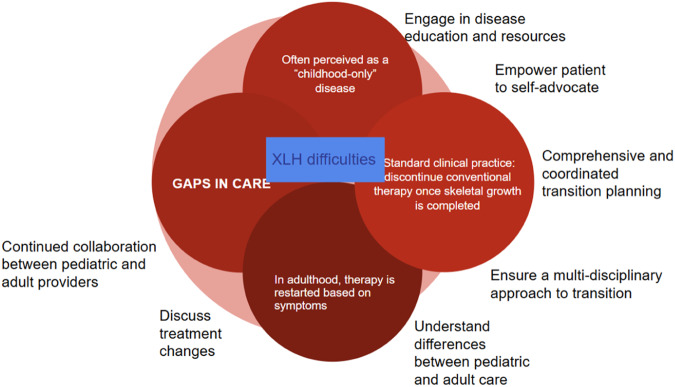
Role of the transition manager

**Fig. 2:**
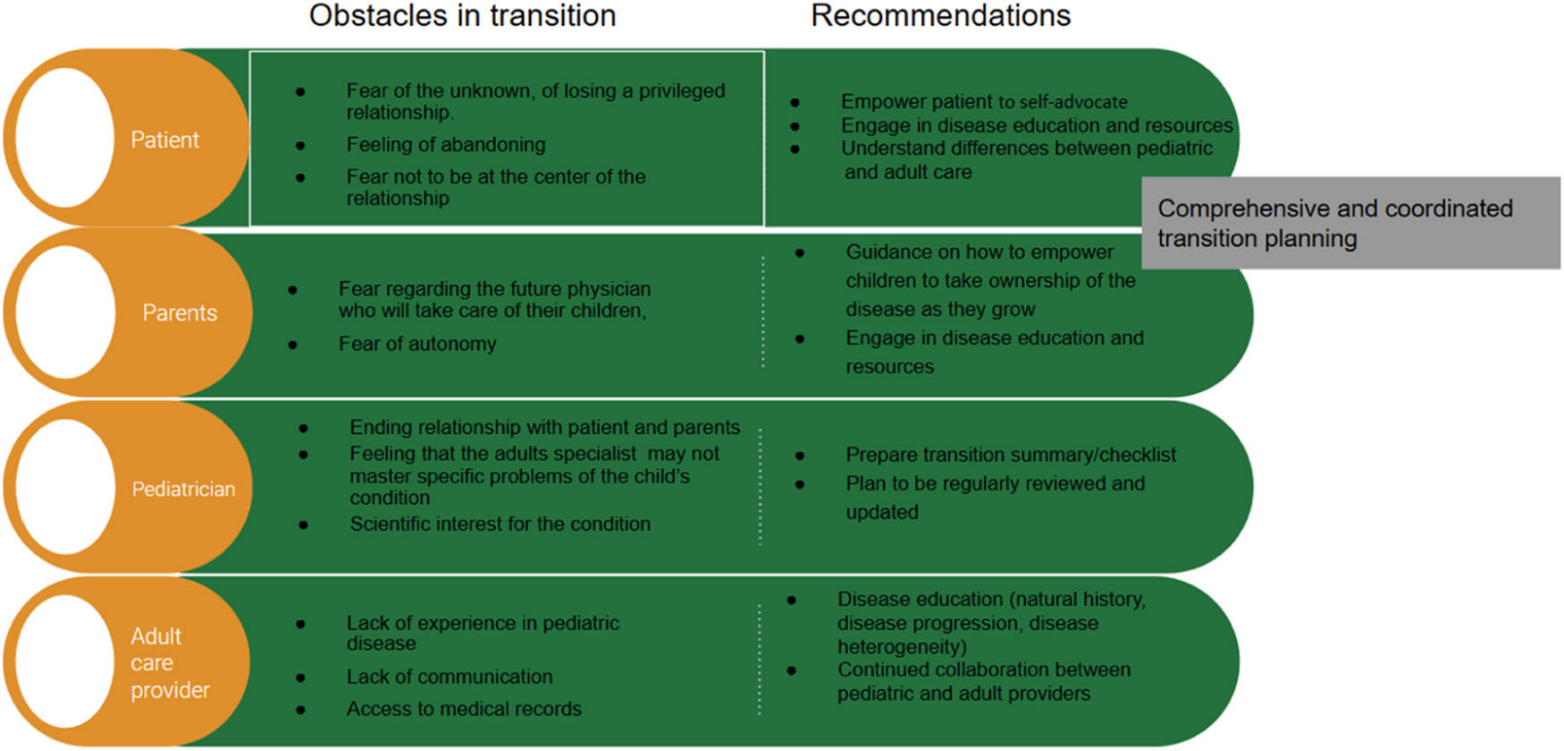
Barriers in the process

The transition Manager should be one of the pillars in the transition. This professional, usually a nurse, facilitates coordination between the different specialists in the same hospital, between different hospitals and with other clinical levels. His/her role is to act as a liaison between specialists, the patient and family, in order to ensure the right healthcare coordination and guided emotional support during the transition. This professional should be part of both hospital teams, pediatric and adult, in order to optimize interdisciplinary work and ensure continuity of care, as well as effective and expedited follow-up [34, 40]. The duties of this professional include [5, 13, 25, 26] (Fig. 1):

- Planning
  - Preparation and management of individual transition plans.
- Running the team
  Interdisciplinary management coordination. Monitoring, evaluation and feedback.
  Creation and coordination of the patient referral team.
  Communication and coordination with the receiving team.
  Coordination with the administrative areas of the respective institutions as part of the transition process.
- Coordination and implementation

- Completion of the transition plan forms, if relevant.
- Complete documentation in the patient’s chart of all the activities carried out by the team.
- Preparation and handover of the clinical record, forms and documents from the pediatric team to the adult care team.
- Identification and management of barriers in the transition process, related to the patient and family, the healthcare professionals, or the administrative area (Fig. 2).

- Systematic assessment of the transition process, extended beyond its completion, with feedback and implementation of improvement plans.
- Evaluation of the transition process.
- Support to the patient and family
- Communication and ongoing support to the patient and family
- Comprehensive education for the patient and family regarding the transition process, the underlying disease and the care of the patient’s health.
- Healthcare-related education (empowerment in relation to treatment, body and symptom awareness; identification of healthcare and social support networks, and counseling regarding adolescence-related topics).
- Fostering the creation of groups of patients and families with the same disease, or promoting participation in support groups for patients with orphan diseases.

### Recommendation 15

**The following is recommended regarding the profile of the case manager for the process of transitioning patients with XLH**

This professional (usually a nurse) should have knowledge of the comprehensive approach to patients with XLH [41]. Depending on the characteristics and resources of the institution, other options are a general practitioner or a social worker [13, 26].

### Recommendation 16

**The following is recommended regarding the frequency of training for the team in charge of transitioning patients with XLH**

- At the time of creating the transition team [25, 27].
- Upon arrival of new members to the team [25, 27].
- In case of change of the coordinator or other members of the transition team [25, 27].
- Every 12 months for the purpose of adjusting processes in accordance with identified needs [25, 27].
- Whenever there is a need for significant changes to the process, the work of the team, or the management, follow-up, and improvement plans [25–27, 42]
- Whenever new relevant information emerges, calling for an update [25–27, 42].

### Recommendation 17

**The following is recommended regarding the frequency with which the content of training for the transition team working with patients with XLH should be updated:**

- The training content should be updated on an annual basis or, alternatively, whenever new relevant medical evidence emerges [25, 43].

### Recommendation 18

**The following is recommended regarding the frequency of training for patients and families on the transition process in XLH**

- Education programs pertaining to specific aspects of the disease and more general health topics and information on the health system are needed in order to help patients achieve full autonomy. They could be carried out according to the following schedule: [25–27, 44, 45].
- At the time of entering the transition program.
- Online or face-to-face lectures every 4 to 6 months by age groups or by transition stage.
- Meetings every 6 months to refresh concepts, reinforce the need for follow-up, and treatment if needed, or to ask specific questions from individual patients.
- At any time in case the need for additional training or in-depth information on a specific topic is identified at the individual or group level.
- At each visit with the transition team.

### Recommendation 19

**The following is recommended in order to inform patients with XLH and their families of the activities of the team during the transition process:**

- A formal meeting with the whole team at the time of initiating the transition process to introduce the program and the participating professionals. The transition manager must also be introduced in order to facilitate communication with the family and build trust around the process [13, 25, 27, 43].
- Follow-up visits for each patient in the transition program in order to update individual activities and to provide continuous training for the patient and the family member [25, 27].
- Different options for conveying information (according to availability and institutional policies): institutional website, brochures, infographics, short update e-mail messages and instant messaging apps, among others [13, 26].
- The frequency varies depending on individual patient needs and characteristics.

### Recommendation 20

**The following is recommended regarding the person in charge of informing patients with XLH and their families about the activities conducted by the team as part of the transition process:**

This task should be the joint responsibility of the leader of the interdisciplinary transition team (endocrinologist, nephrologist) and the transition manager [13, 25–27].

### Recommendation 21

**The following is recommended regarding the timing for informing patients with XLH and their families about the activities conducted by the team as part of the transition process:**

- At the initiation of the transition process, introducing the program and the participating professionals, in order to inform the patient about the minimum requirements associated with the process [13, 25, 46].
- During the visits scheduled as part of the transition process for each patient, in order to provide updates [16, 25].
- Plan a meeting every 12 months with each patient to provide information about their individual process.
- The frequency and number of informative and educational meetings can vary depending on individual patient needs and characteristics. It is important to consider the possibility of additional meetings in case there is a need to provide information about new developments, significant changes regarding the treatment, or the transition process itself [13, 25].

### Recommendation 22

**The following is recommended regarding the assessments required during each follow-up visit in terms of clinical, paraclinical and imaging findings once the pediatric patient with XLH initiates the transition process:**

Treatment monitoring, clinical and laboratory findings, radiological, dental, neurosurgical, rheumatological, cardiovascular, renal and audiological assessment [5, 6, 13, 31, 47]

- Clinical considerations
  Thorough clinical history and physical examination: Weight, height, body mass index, blood pressure, growth velocity, pubertal development, presence of pain, lower limb axis and deformities, with intercondylar and intermalleolar distance measurement, and functional capacity
  Symptoms, disease complications (headache, dental disease, maxillofacial cellulitis, periodontal disease, musculoskeletal pain, pseudofractures, fatigue, depressive or emotional symptoms)
  Treatment adherence, tolerance, efficacy perception and adverse effects
  Updated interdisciplinary assessments.
- Paraclinical considerations
  - Laboratory tests:
  In serum: Phosphorus, calcium, parathormone hormone, alkaline phosphatase, creatinine, 25 OH vitamin D and 1,25 OH vitamin D.
  In urine: Urinalysis, calcium, phosphorus and creatinine (in urine, isolated sample)
- Imaging

Based on the individualized assessment, ask for a renal ultrasound and long bone X-rays (general, no specific time interval).

### Recommendation 23

**The following is recommended regarding the assessment of psychosocial factors during every follow-up visit, once pediatric patients with a diagnosis of XLH initiate the transition process:**

- At each follow-up visit, assess all aspects that can either hinder or ease the transition to adult life, including the degree of overprotection. Additionally, psychosocial and socio-familial questionnaires should be used to gather information regarding family, financial or other issues that might impact the transition process [13, 26, 27, 46, 47].
- Patient emotional and cognitive maturity, self-care capability, autonomy and independence.
- Knowledge of the disease, laboratory tests and treatment (dose, ability to obtain the medication, what to do in case of adverse reactions or skipping a dose, etc.).
- Adherence to treatment.
- Attendance to appointments (can patients take responsibility for obtaining a follow-up appointment, attending appointments on their own, asking for an appointment in case of changes in their health condition).
- Ability to communicate and ask questions of healthcare professionals.
- Performance at school and/or work.
- Self-perception and perception of their own health. Self-esteem.
- Functionality of the family and of the support networks. Interactions with peers or orphan disease support groups.
- Patient and family opinion regarding the transition process. Level of knowledge regarding the process.
- During the visit, questionnaires can be used to determine patient readiness. These include the Transition Readiness Assessment Questionnaire (TRAQ) 5.0, validated in Argentina (includes 20 items in 5 sub-scales: medication, appointment attendance, monitoring of health problems, communication with healthcare professionals and coping with activities of daily living). This is a self-administered tool and each item is scored using a Likert scale from 1 to 5 (1 being minimum autonomy and 5 being maximum autonomy [25, 26].

### Recommendation 24

**The following is recommended regarding the maximum interval between follow-up visits for patients with XLH who are in the transition process:**

This aspect has not been clearly defined in the literature. However, it may vary significantly depending on patient and family profile and needs [13, 25]. Follow-up frequency in patients with XLH must be individualized. Children and adolescents in a rapid growth phase or with changes in medication dose or treatment type should be reassessed every 3 months. Patients with stable clinical and laboratory findings and no therapeutic changes should be reassessed every 6 months [13, 25].

### Recommendation 25

**The following is recommended regarding the minimum number of follow-up visits in patients with XLH who are in the transition process:**

- It depends on the course of the disease and patient and family response to the transition process. [13, 25].
- In the event the medical team in charge of the transition is the main treating team, 3 visits in the year are recommended, depending on the time of initiation of the transition process [5].

### Recommendation 26

**The following is recommended regarding the requirements to determine that the transition process has come to an end in patients with XLH:**

- Acknowledgment of the chronicity of the disease [25, 26, 34].
- Patient autonomy and empowerment [25, 26, 34].
- Pharmacological treatment and adjunct therapies not in the process of changing [25, 26, 34].
- Adherence to treatment [25, 26, 34].
- Follow-up attendance and a thorough and adequate assessment by the receiving team [25, 26, 34].
- Patient in stable clinical and metabolic condition [25, 26, 34].
- Absence of neurological disability or cognitive decline [25, 26, 34].

### Recommendation 27

**The following is recommended regarding the circumstances in which the final visit to close the transition process in patients with XLH should be postponed:**

- Clinical deterioration or development of acute complications [13, 25].
- Need to change the treatment [13].
- Non-adherence to the indications and management plan proposed by the receiving team [25].
- Evidence of incomplete or inadequate adaptation to the new treating team by the patient, the family or the transition team [25].
- Family or personal patient dysfunction, with negative impact on adaptation during the transition process [25].
- Absence of patient autonomy, empowerment or acknowledgement of the disease [13, 25].

### Recommendation 28

**Regarding verification of adequate adaptation to the adult service by patients with X-linked hypophosphatemia who have completed the transition process, the recommendation is to carry out the process in the following way:**

- Telephone call after the first appointment with the adult treating team [25].
- Follow-up by phone according to the characteristics and needs of the patient and family [48].
- Follow-up visit 6 months to 1 year after transfer to the adult service, including assessment of the following: [25, 26].
- Self-care capability and adherence to treatment.
- Quality of life (anxiety, isolation, self-esteem and self-perception of the health condition).
- Patient perspectives regarding the results of the treatment.
- Questions/satisfaction surveys for the patient and family regarding their adaptation to the process [25].
- Review of the opinion of the receiving treating team regarding patient attitude and adaptation to the process [25].
- Oversight of attendance to appointments and follow-up visits with the receiving team, and adherence to treatment [25].

### Recommendation 29

**The following is recommended regarding information about patients with XLH that should be shared among services involved in the transition process:**

- Complete information about medical care provided in the pediatric hospital service shared with the adult team that will take over the care of the patient in the future [13, 14, 25].
- Patient identification information.
- Thorough medical history.
- Date of XLH diagnosis.
- Description of the disease and comorbidities.
- Time on follow-up.
- Course of the disease.
- Clinical and paraclinical assessment.
- Treatments received: Date of initiation and discontinuation, dosing, adverse effects, tolerance, efficacy, adherence.
- Complications associated with the underlying disease or the medications.
- Interdisciplinary assessment reports.
- Appointments for interdisciplinary assessments and pending paraclinical tests.
- Family and social history, and report.
- Patient schooling level.
- Risky behaviors.
- Pattern of coping with the disease and treatment.
- Level of independence, autonomy and self-care.
- Any other relevant information that the transition manager or the pediatric treatment group considers important about the patient or relevant for the transition.

### Recommendation 30

**The following is recommended regarding the indicators that could be considered for reviewing the performance of the team and the impact of the transition process on patients with XLH:**

- Quality indicators based on the proposed goals, and objective and reproducible measurements: [13, 25, 26, 49–52]
- Percentage of patients who complete the entire transition process.
- Percentage of patients who continue on adequate follow-up with the receiving group for a period of time.
- Patient and family perception of the care experience (self-care capability).
- Visual analog scale to measure patient and family satisfaction with the transition process
- Physician opinion survey regarding medical care transition (use of the transition plan designed with the team, understanding and achievement of the proposed goals, attendance and compliance with the proposed objectives).
- Indicator of attendance to scheduled appointments.
- Commitment and adherence to pharmacological treatment and management by the receiving team.

### Recommendation 31

**The following is recommended regarding the timing for assessing compliance with the objectives of the transition process for patients with XLH:**

- Regular assessments to determine compliance with objectives by the transition team, the patient and the family during the different stages of the process, documenting achievement of the proposed goals [5, 25, 26, 51].
- Assessments are suggested at the time of the closure visit and 12 months afterwards [25].

### Recommendation 32

**The following is recommended regarding documentation of the assessment process to determine compliance with the objectives of the transition in patients with XLH:**

**Fig. 3:**
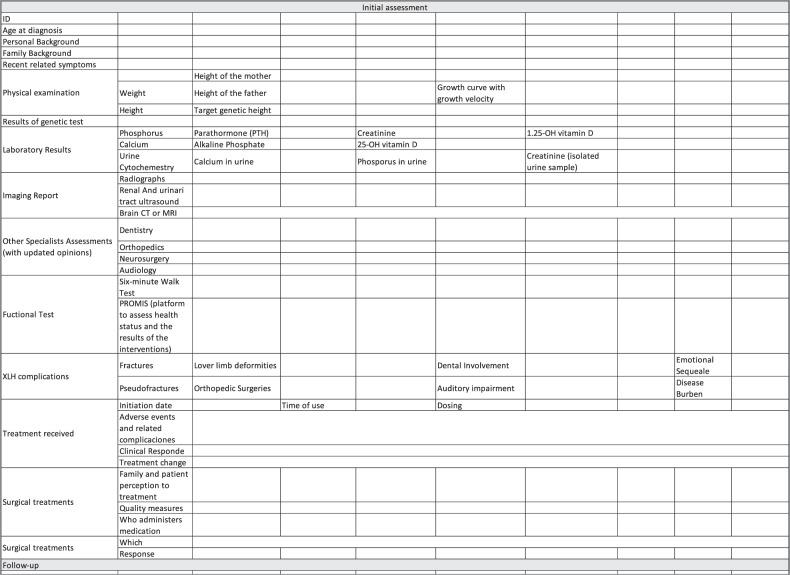
Checklist for patients with XHL during the transition

Design and use of tools and checklists (Fig. 3) for the transition team, the patient and the family: [13, 25, 26, 53].

- Documenting checklists of proposed and performed activities, and of adequate compliance.
- Completion of review and verification lists of key activities at each point in the process and at the end thereof: number of consultation visits, time required to achieve the objectives, follow-up at the end of the transition, and percentage of patients lost to follow-up.
- Completion and documentation of the related quality indicators.

### Recommendation 33

**The following is recommended regarding the minimal requirements that have to be met by the health system in order to ensure the development of an adequate transition process in patients with XLH:**

- Ensure: [13, 25, 26, 53].
- Complete funding for the transition clinical unit in order to allow its comprehensive development.
- Specialists who participate in the transition process in the same institution or connected in such a way as to allow patient care to flow seamlessly.
- Adoption of updated scientific recommendations regarding the care of patients with XLH.
- Availability of physical facilities to conduct the process, including meetings, planning, execution, and joint pediatric and adult consultations.
- Adequate availability and coverage to allow easy access to the health system for patients, diagnostic tests, interdisciplinary assessments and comprehensive treatment.
- Checklist for patients with XHL during transition.

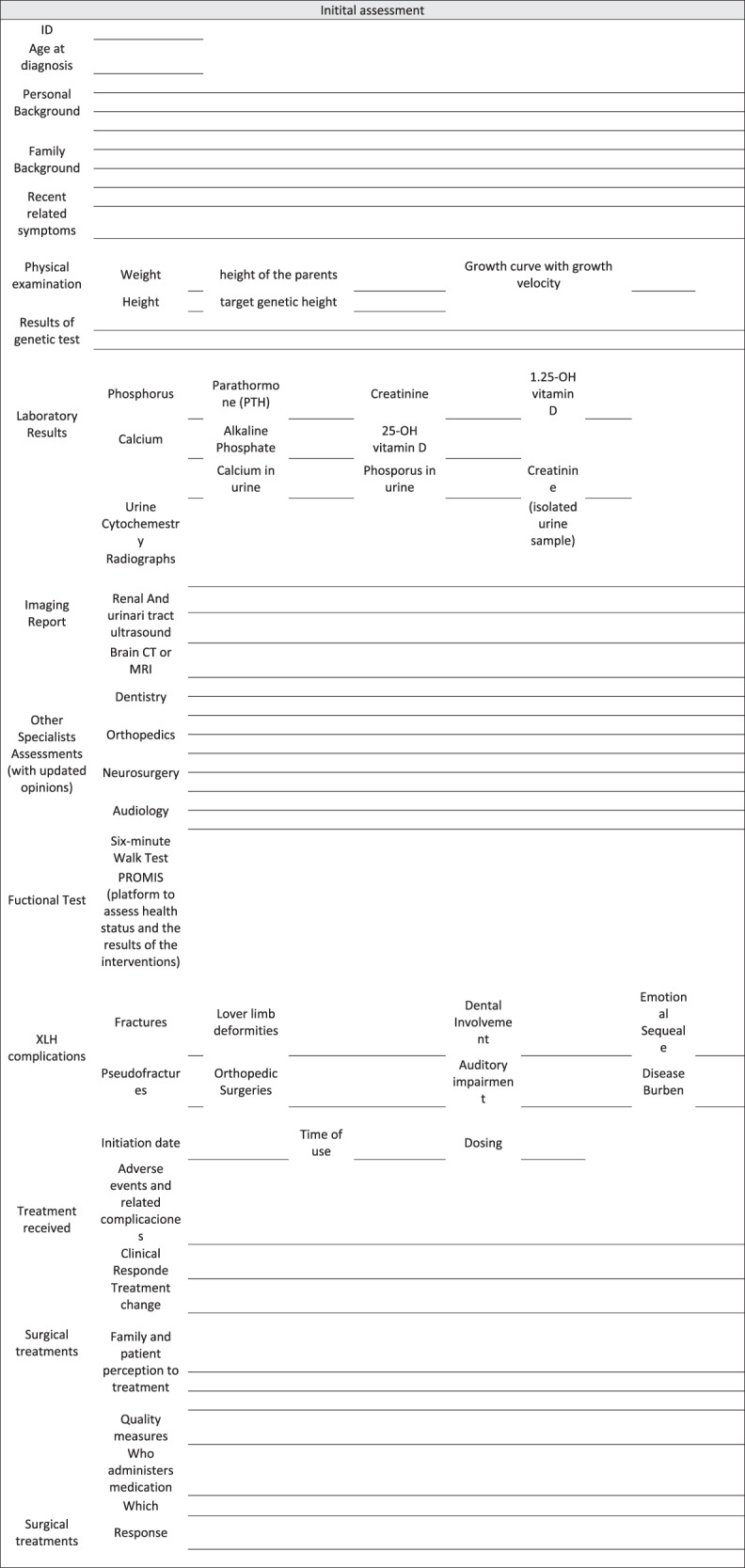
 
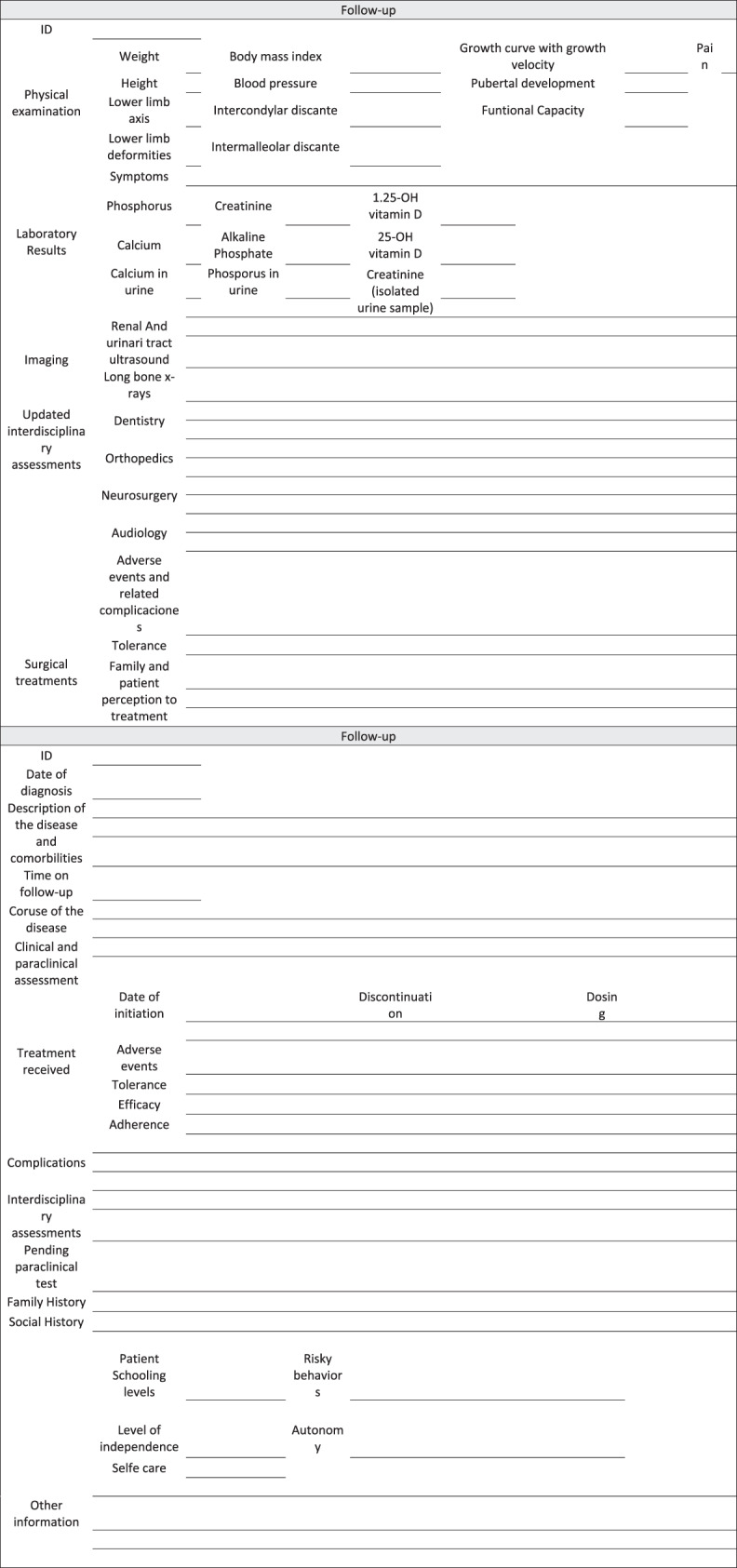


## Conclusions

The transition into the adult life of patients with chronic diseases associated with morbidity and mortality, as is the case of XLH, is a complex process. The essential pillar in managing these patients must not be limited to medical intervention and care but should involve a comprehensive approach that includes psychosocial components of adult life as well as education/vocation. This requires a multidisciplinary team trained in this process (14).

Programs of transition to adult life must be tailored to the needs of the individual patients, within the health system of which patients are a part.

This article offers a set of recommendations for the Latin-American context. The 33 recommendations are designed to help health institutions and providers who manage patients with XLH, as well as patients and families, to accomplish a successful transition into adult life, working from a multidisciplinary and comprehensive approach.

### Supplementary information

Annex A and B

## Data Availability

Data sharing does not apply to this article as no datasets were generated or analyzed during the current study.
