# Supplementary material for: Latin-American consensus on the transition into adult life of patients with X-linked hypophosphatemia
Source: Endocrine. 2023 Dec 20;84(1):76–91. doi: 10.1007/s12020-023-03624-z (PMC10987342; doi:10.1007/s12020-023-03624-z)
Supplement: Supplementary file 1 — Annex A and B [file 12020_2023_3624_MOESM1_ESM.docx]

Annex A.

| **Questions** |
| --- |
| 1. Elementos facilitadores - Apoyo institucional (Enablers - Institutional support)  ¿Cuál es el nivel mínimo de atención o complejidad que deben tener las instituciones en las que se implementen planes de transición para pacientes con hipofosfatemia familiar ligada a X?  What is the minimum level of care or complexity that institutions implementing transition plans for patients with familial X-linked hypophosphatemia should have? |
| 2. Elementos facilitadores - Previsión del tiempo necesario para preparar la transición (Enablers - Forecast of the time needed to prepare for the transition)  ¿Cuáles son los factores psicosociales del paciente con hipofosfatemia familiar ligada a X que se deben tener en cuenta para determinar el momento adecuado para iniciar el proceso de transición? (definir puntos de corte, si aplica)  What are the psychosocial factors of the patient with familial X-linked hypophosphatemia that should be considered to determine the appropriate time to initiate the transition process (define cut-off points, if applicable)? |
| 3. Elementos facilitadores - Previsión del tiempo necesario para preparar la transición (Enablers - Forecast of the time needed to prepare for the transition)  ¿Cuáles son los criterios clínicos del paciente con hipofosfatemia familiar ligada a X (desagregados) que se deben tener en cuenta para determinar el momento adecuado para iniciar el proceso de transición? (definir puntos de corte, si aplica)  What should clinical criteria for the patient with familial X-linked hypophosphatemia (disaggregated) be considered to determine the appropriate time to start the transition process (define cut-off points, if applicable)? |
| 4. Elementos facilitadores - Previsión del tiempo necesario para preparar la transición (Enablers - Forecast of the time needed to prepare for the transition)  ¿Cuál es el tiempo promedio necesario para el proceso de transición para pacientes con hipofosfatemia familiar ligada a X?  What is the average time needed for the transition process for patients with familial X-linked hypophosphatemia? |
| 5. Elementos facilitadores - Implicación del equipo asistencial (Enablers - Care team involvement)  ¿Cuáles son los profesionales que se deben involucrar en el proceso de transición para los pacientes con hipofosfatemia familiar ligada a X?  Which professionals should be involved in the transition process for patients with familial X-linked hypophosphatemia? |
| 6. Elementos facilitadores - Implicación del equipo asistencial (Enablers - Care team involvement)  ¿Cuáles serían los profesionales que, como mínimo, se deben hacer parte del equipo institucional de transición para los pacientes con hipofosfatemia familiar ligada a X?  Which professionals, at a minimum, should be part of the institutional transition team for patients with familial X-linked hypophosphatemia? |
| 7. Elementos facilitadores - Organización del trabajo (Enablers - Work organization)  ¿Con qué periodicidad se debe reunir el equipo para evaluar y planear las acciones del proceso de transición de los pacientes activos?  How often should the team meet to evaluate and plan actions for the active patient transition process? |
| 8. Análisis de la situación - Evaluación inicial (Situation analysis - Initial evaluation)  ¿Cuáles deben ser los profesionales que participen a la evaluación inicial del proceso de transición?  Which professionals should be involved in the initial evaluation of the transition process? |
| 9. Análisis de la situación - Evaluación inicial (Situation analysis - Initial evaluation)  ¿Cuáles deben ser los contenidos clínicos de la evaluación inicial del proceso de transición?  What should be the clinical contents of the initial evaluation of the transition process? |
| 10. Análisis de la situación - Evaluación inicial (Situation analysis - Initial evaluation)  ¿Cuáles deben ser los contenidos psicosociales de la evaluación inicial del proceso de transición?  What should be the psychosocial contents of the initial evaluation of the transition process? |
| 11. Análisis de la situación - Evaluación inicial (Situation analysis - Initial evaluation)  ¿Cuáles son los aspectos a evaluar en relación con el esquema terapéutico que viene recibiendo el paciente, durante la evaluación inicial del proceso de transición?  What aspects are to be evaluated concerning the therapeutic scheme that the patient has been receiving during the initial evaluation of the transition process? |
| 12. Análisis de la situación - Evaluación inicial (Situation analysis - Initial evaluation)  ¿En qué situaciones considera que se requiere consultoría genética?  In which situations do you consider that genetic consulting is required? |
| 13. Análisis de la situación - Evaluación inicial (Situation analysis - Initial evaluation)  ¿En qué situaciones que se requiere solicitar prueba molecular?  In what situations is it required to request molecular testing? |
| 14. Análisis de la situación - Profesional gestor de la transición (Situation analysis - Professional transition manager)  ¿Cuáles deben ser las funciones del gestor de transición en un programa de transición para pacientes hipofosfatemia familiar ligada a X?  What should be the roles of the transition manager in a transition program for familial X-linked hypophosphatemia patients? |
| 15. Análisis de la situación - Profesional gestor de la transición (Situation analysis - Professional transition manager)  Para el proceso de transición de los pacientes con hipofosfatemia familiar ligada a X, ¿cuál es el perfil profesional del gestor de transición?  For the transition process of patients with familial X-linked hypophosphatemia, what is the professional profile of the transition manager? |
| 16. Análisis de la situación - Actividades de formación y capacitación de los profesionales de la salud y de los pacientes (Situation analysis - Education and training activities for health care professionals and patients)  ¿Con qué periodicidad se deberían hacer capacitaciones para el equipo de transición para pacientes con hipofosfatemia familiar ligada a X?  How often should there be training for the transition team for patients with familial X-linked hypophosphatemia? |
| 17. Análisis de la situación - Actividades de formación y capacitación de los profesionales de la salud y de los pacientes (Situation analysis - Education and training activities for health care professionals and patients)  ¿Con qué periodicidad debería actualizarse el contenido de estas capacitaciones?  How often should the content of these pieces of training be updated? |
| 18. Análisis de la situación - Actividades de formación y capacitación de los profesionales de la salud y de los pacientes (Situation analysis - Education and training activities for health care professionals and patients)  ¿Con qué periodicidad se deberían hacer capacitaciones para los pacientes y sus familias en el proceso de transición de hipofosfatemia familiar ligada a X?  How often should there be training for patients and their families in the transition process of familial X-linked hypophosphatemia? |
| 19. Análisis de la situación - Actividades de transferencia de información (Situation analysis - Information transfer activities)  ¿Cómo se debería informar al paciente y a su familia sobre las actividades realizadas por el equipo dentro del proceso de transición?  How should the patient and family be informed about the activities carried out by the team within the transition process? |
| 20. Análisis de la situación - Actividades de transferencia de información (Situation analysis - Information transfer activities)  ¿Quién debería ser el responsable de informar a los pacientes y a sus familias sobre las actividades realizadas por el equipo dentro del proceso de transición?  Who should be responsible for informing patients and their families about the activities carried out by the team within the transition process? |
| 21. Análisis de la situación - Actividades de transferencia de información (Situation analysis - Information transfer activities)  ¿En qué momento se debería informar a los pacientes y a sus familias sobre las actividades realizadas por el equipo dentro del proceso de transición?  At what point should patients and their families be informed about the activities carried out by the team within the transition process? |
| 22. Seguimiento del paciente - Consultas de transición (Patient follow-up - Transition consultations)  Una vez el paciente en cuidado pediátrico con diagnóstico de hipofosfatemia familiar ligada a X inicia el proceso de transición, ¿qué debe evaluarse, en cada consulta de seguimiento, en relación con los aspectos clínicos, paraclínicos e imagenológicos?  Once the patient in pediatric care with a diagnosis of familial X-linked hypophosphatemia starts the transition process, what should be evaluated, at each follow-up visit, about clinical, paraclinical, and imaging aspects? |
| 23. Seguimiento del paciente - Consultas de transición (Patient follow-up - Transition consultations)  Una vez el paciente en cuidado pediátrico con diagnóstico de hipofosfatemia familiar ligada a X inicia el proceso de transición, ¿qué debe evaluarse, en cada consulta de seguimiento, en relación con los aspectos psicosociales?  Once the patient in pediatric care with a diagnosis of familial X-linked hypophosphatemia begins the transition process, what should be evaluated about psychosocial aspects at each follow-up visit? |
| 24. Seguimiento del paciente - Consultas de transición (Patient follow-up - Transition consultations)  ¿Cuál sería el lapso máximo de separación entre las consultas de seguimiento para pacientes con hipofosfatemia familiar ligada a X que se encuentran en proceso de transición?  What would be the maximum length between follow-up visits for patients with familial X-linked hypophosphatemia undergoing transition? |
| 25. Seguimiento del paciente - Consultas de transición (Patient follow-up - Transition consultations)  ¿Cuál sería el número mínimo de consultas de seguimiento que deberían realizarse en pacientes con hipofosfatemia familiar ligada a X que se encuentran en proceso de transición?  What would be the minimum number of follow-up visits that should be performed in patients with familial X-linked hypophosphatemia who are undergoing transition? |
| 26. Seguimiento del paciente - Consulta de cierre del proceso de transición (Patient follow-up - Closing consultation of the transition process.)  ¿Cuáles son los criterios para determinar que se debe realizar la consulta de cierre del proceso de transición en pacientes con hipofosfatemia familiar ligada a X?  What are the criteria for determining that transition closure consultation should be performed in patients with familial X-linked hypophosphatemia? |
| 27. Seguimiento del paciente - Consulta de cierre del proceso de transición (Patient follow-up - Closing consultation of the transition process.)  ¿En qué circunstancias se debería postergar la realización de la consulta de cierre del proceso de transición en pacientes con hipofosfatemia familiar ligada a X?  Under what circumstances should a transition closure consultation in patients with familial X-linked hypophosphatemia be postponed? |
| 28. Seguimiento del paciente - Seguimiento del paciente postransición (Patient follow-up - Post-transition patient follow-up)  En pacientes con hipofosfatemia familiar ligada a X que han realizado el proceso de transición, ¿cómo debería llevarse a cabo la verificación de la adecuada adaptación al servicio de adultos?  In patients with familial X-linked hypophosphatemia who have undergone the transition process, how should adequate adaptation to adult service be verified? |
| 29. Actividades de soporte y documentación (Support and documentation activities)  ¿Qué información de los pacientes con hipofosfatemia familiar ligada a X debería ser compartida entre servicios que forman parte del proceso de transición?  What should information from patients with familial X-linked hypophosphatemia be shared between services that are part of the transition process? |
| 30. Evaluación de calidad del proceso de transición (Transition process quality assessment)  ¿Qué indicadores podrían considerarse para evaluar el desempeño del equipo y el impacto del proceso de transición para pacientes con hipofosfatemia familiar ligada a X?  What indicators could be considered to evaluate team performance and the impact of the transition process for patients with familial X-linked hypophosphatemia? |
| 31. Evaluación de calidad del proceso de transición (Transition process quality assessment)  ¿En qué momento debería llevarse a cabo la evaluación del cumplimiento de los objetivos del proceso de transición para pacientes con hipofosfatemia familiar ligada al X?  When will an assessment of compliance with the transition process goals for patients with familial X-linked hypophosphatemia be conducted? |
| 32. Evaluación de calidad del proceso de transición (Transition process quality assessment)  ¿Cómo debería documentarse el proceso de evaluación del cumplimiento de los objetivos del proceso de transición para pacientes con hipofosfatemia familiar ligada al X?  How should the process of assessing compliance with the transition process goals for patients with familial X-linked hypophosphatemia be documented? |
| 33. Sistema de salud (Health system)  ¿Cuáles serían los requisitos mínimos que debe cumplir el Sistema de Salud para garantizar el desarrollo de un adecuado proceso de transición en un paciente con diagnóstico de XLH?  What are the minimum requirements that the Health System must comply with to guarantee the development of an adequate transition process in a patient diagnosed with XLH? |

Annex B

Recommendations table

| **Recommendation 1**  **The following is recommended regarding the minimum level of care or complexity required in institutions that implement plans for transitioning patients with XL** | - A long-term care plan which foresees transition to adult outpatient care and requires a level III healthcare service.^5,12,13,26-29^ - Adequate multidisciplinary management led by an expert in metabolic bone disorders.^5,13,26-2^ |
| --- | --- |
| **Recommendation 2 The following is recommended regarding the psychosocial factors of patients with XLH which need to be considered to determine the right timing to initiate the transition process:** | - Consider the age of 12 years as the appropriate age to start working with the patient and family on the considerations related to the transition, given that the child is sufficiently ready to understand the process and become actively involved. - Regarding the assessment of the psychosocial status, the recommendation is to consider the following factors:^13,25-30^   - The patient’s psychological and emotional maturity.   - The patient’s perception and ability to plan and perform self-care activities.   - The cognitive ability of the patient and caregivers.   - Patient and family ability to acquire and integrate information on the disease and on the indicated medical management and care, and to incorporate it into daily life; the ability to identify problems in the clinical course of the disease and communicate them to the treating physician adequately and on a timely basis.   - Family, personal, school and work support networks and their adequate functioning.   - Cognitive disability or severe psychiatric disorder in the patient or the family/caregiver.   - Sociocultural level of the patient and family.   - The local health system and the capacity to offer an adequate transition of medical care between the pediatric age and adulthood, and the patient’s ability to adapt to this change. |
| **Recommendation 3:**  **The following clinical criteria are recommended in patients with XLH (disaggregated) in order to determine the right timing for the initiation of the transition process:** | - Disease stability:^5,13,26,31^   - Adequate pain control   - Stabilization of fractures and pseudofractures   - No need for orthopedic surgery in the immediate future   - Updated tests and growth curves   - Stabilization of the calcium-phosphorus metabolism (alkaline phosphatase, parathormone, serum calcium and phosphorus, and calciuria)   - Mobility to allow self-care. - Treatment stability, with no recent medication or therapy changes^5,13,26,31^ - Absence of complications or clinical deterioration in the past few months^5,13,26,3^ |
| **Recommendation 4:**  **The following is recommended regarding the average time for the process of transitioning patients with XLH:** | - The length of the transition period should vary depending on individual patient characteristics. It begins in the pediatric age, at around 12 years, when additional information is started to be provided with the aim of promoting autonomy, and ideally extends until 18-21 years of age. (This limit can be extended when needed, regardless of the local administrative criteria).^5,13,25,27,33^ - The average varies depending on the time when the process is initiated but requires at least 2-3 years and may last up to 6-9 years.^25-27,30,33^ |
| **Recommendation 5:**  **The following is recommended regarding the professionals that should be involved in the transition process of patients with XLH:** | - Involve specialists in pediatrics and internal medicine (endocrinology and nephrology) in the process of transitioning patients with XLH to act as leaders of the referring and receiving teams.^13,27,30^ - Use a targeted and individualized interdisciplinary approach, working with other specialties and professions such as pediatrics, internal medicine, genetics, orthopedics, psychiatry, neurosurgery, otolaryngology, physical medicine and rehabilitation, pain management specialists, psychology, nursing, dentistry, nutrition and dietetics, physical therapy, occupational therapy, ophthalmology, and audiology, depending on individual patient characteristics.^5,26^ - The process should be coordinated with the administrative area of the healthcare institutions involved, through case managers or social workers.^11,26-28^The process should be coordinated with the administrative area of the healthcare institutions involved, through case managers or social workers. |
| **Recommendation 6:**  **It is recommended that at least the following professionals are part of the institutional transition team for patients with XLH:** | - An expert in XLH from the pediatric area (pediatric endocrinologist or nephrologist) and his/her counterpart in adult medicine (endocrinologist or nephrologist) who act as leaders of the “referring and receiving” teams.^1,13,26,27^ - A physician, a nurse, a social worker (with experience in transitioning patients with chronic diseases), clinical professionals, volunteers, and administrative staff.^1,13,26^ |
| **Recommendation 7:**  **The following is recommended regarding the frequency with which the team must meet to assess and plan actions in the process of transitioning active patients with XLH:** | - It should vary depending on the number of patients who are active in the transition program and their stage in the process. - Convene the team every 4 months in average. Frequency can vary depending on the local situation in each country. - Hold one meeting every 3-4 months during the transition period ^25,27^ - Build the transition process during the pediatric stage as part of patient education. It must be intensified starting at 12 years of age to build autonomy conditions for the patient and to enable the pediatric team to prepare appropriately for severing the bond with the patient. Finally, the family should abstain from participating actively in the care of the patient.^27^ - Refer the patient from one place to another for future management in hospitals with pediatric and adult services, holding online meetings that can complement face-to-face encounters to make the transition process easier. Online meetings are particularly important in those cases in which transition implies going to a different hospital than the one providing care during the pediatric age.^25^ |
| **Recommendation 8**  **The following is recommended regarding professionals who participate in the initial assessment of the transition process for patients with XL** | - An expert specialized in XLH from the pediatric area, such as pediatric endocrinologist or nephrologist and his/her adult medicine counterpart such as an endocrinologist or nephrologist. Ideally, they should be accompanied by nursing, social work and mental health professionals.^13,25,27,34^ - The person who will act as general coordinator (endocrinologist and/or nephrologist) of the care in the adult hospital, as well as the case manager, should be designated from the start of the process, the use of a care manager arises from the shortage of pediatricians or adult care specialists in some hospital in our region; providing a transversal role in care from the care manager as a key element in countries with heterogeneous health care systems.^13,25^ |
| **Recommendation 9:**  **The following is recommended regarding the clinical content of the initial assessment carried out as part of the transition process in patients with XLH:** | - A list of clinical data, including potential complications and the established management for the patient^5,13,26,27,35^, as follows:   - Patient identification information   - Age at the time of diagnosis   - Complete personal background history (emphasis on the surgical background)   - Complete family background   - Recent related symptoms   - Complete physical examination including weight, height, height of the parents, target genetic height.   - Growth curve with growth velocity   - Result of the genetic test for XLH   - Laboratory results at the time of diagnosis and of the most recent tests. In serum: phosphorus, calcium, parathormone (PTH), alkaline phosphatase, creatinine, 25 OH vitamin D and 1.25 OH vitamin D. In urine: urine cytochemistry, calcium, phosphorus, creatinine (in isolated urine sample).   - Imaging report: radiographs, renal and urinary tract ultrasound, brain CT or MRI (if available)   - Assessments by other specialists: dentistry, orthopedics, neurosurgery, audiology, with updated opinions   - Functional tests: Six-minute walk test, Promis (platform to assess health status and the results of the interventions), if available and/or if it has been performed.   - XLH-related complications: Fractures, pseudofractures, lower limb deformities, orthopedic surgeries, dental involvement, auditory impairment, emotional sequelae, and disease burden   - Treatments received: Time of use, adherence, clinical response and related complications (nephrocalcinosis and/or hyperparathyroidism)   - Surgical treatments received and response to those treatments |
| **Recommendation 10**  **The following is recommended regarding the psychosocial content of the initial assessment in the process of transitioning patients with X-linked hypophosphatemia:** | - Basic family history: Family structure, roles and basic functioning^13,27,35^ - Diagnosis of cognitive impairments or psychiatric diseases in the patient or family members/caregivers^13,27,36^ - Adherence to management and follow-up. In case they are inadequate, the reasons should be identified^13,26,27,36^ - Knowledge the patient has regarding the disease, its genetic origin, its chronicity, and the benefit of the treatment. - The patient’s emotional and psychological maturity: Patient autonomy and ability to provide self-care; education level^6,13,27,36,37^ - History of support networks for the patient: Presence and contact between the patient and family with XLH groups or organizations (such as support programs for patients with orphan diseases)^13,27,36^ - Patient and family opinion regarding the initiation of the transition plan, including expectations, barriers and doubts, among other things^13,26,27,36^ |
| **Recommendation 11:**  **During the initial assessment in the transition process, it is recommended to evaluate the following aspects related to the therapeutic regimen currently received by the patient with XLH:** | - Individualized treatments received: Calcitriol, phosphate salts or monoclonal antibodies.^12,13^   - Initiation date   - Dosing   - Time of use   - Adverse effects and related complications   - Adherence to treatment. If inadequate, identify the reasons.   - Assessment of potential treatment changes (in an attempt to improve adherence) or the need to initiate treatment, in case it was not initiated. In the event of treatment change, state the reason.   - Patient and family perception regarding the treatment and its efficacy - Evaluation by means of quality measures (KPIs) in accordance with pharmacovigilance indicators. For example: Lack of therapeutic efficacy, complications associated with the therapeutic regimen, drug-related allergies and adherence to treatment. - It is important to determine who is responsible for administering the medication (the patient or the guardian) and assess adherence to treatment based on clinical symptoms and laboratory tests (alkaline phosphatase and serum phosphorus), as well as patient knowledge regarding the pharmacological treatment (name, dose, schedule, form of administration, side effects).^12,13^ |
| **Recommendation 12:**  **It is recommended to consider genetic counseling for patients with XLH.** | - According to Dahir (2022), genetic counseling must always be offered to patients with X-linked hypophosphatemia at the time of diagnosis.^5,13,25,26,39,40^ - Genetic assessment must be requested during the transition process (if not done previously) in case of diagnostic doubt, to provide counseling and also expand tests to family members, or for counseling the patient regarding disease transmission (families planning pregnancies or pregnant patients).^5,13,25,26,39,40^ |
| **Recommendation 13:**  **It is recommended to ask for molecular tests in patients with XLH:** | In all cases, if not done during the pediatric age. The team in charge of the transition, attended by the professionals who will continue management during adult life, should be responsible for performing the genetic study.^5,13^ It should be noted that genetic testing availability can differ between countries, and thus the recommendation may not be fulfilled when the patient transitions; still preforming genetic testing should be done in the future.  ***Note***: The implementation of this recommendation may be subject to the guidelines, management protocols, consensus and health system characteristics and regulations in each individual country. Specifically in Argentina, having a molecular genetic test is not a prerequisite to initiating treatment |
| **Recommendation 14:**  **The following is recommended regarding the role of the transition manager (or case manager) in a transition program for patients with XLH:** | The transition Manager should be one of the pillars in the transition. This professional, usually a nurse, facilitates coordination between the different specialists in the same hospital, between different hospitals and with other clinical levels. His/her role is to act as a liaison between specialists, the patient and family, in order to ensure the right healthcare coordination and guided emotional support during the transition. This professional should be part of both hospital teams, pediatric and adult, in order to optimize interdisciplinary work and ensure continuity of care, as well as effective and expedited follow-up.^35,41^ The duties of this professional include^5,13,25,2^   - Planning - Preparation and management of individual transition plans - Running the team - Interdisciplinary management coordination. Monitoring, evaluation and feedback - Creation and coordination of the patient referral team - Communication and coordination with the receiving team - Coordination with the administrative areas of the respective institutions as part of the transition process - Coordination and implementation - Completion of the transition plan forms, if relevant - Complete documentation in the patient’s chart of all the activities carried out by the team. - Preparation and handover of the clinical record, forms and documents from the pediatric team to the adult care team - Identification and management of barriers in the transition process, related to the patient and family, the healthcare professionals, or the administrative area - Systematic assessment of the transition process, extended beyond its completion, with feedback and implementation of improvement plans. - Evaluation of the transition process - Support to the patient and family - Communication and ongoing support to the patient and family - Comprehensive education for the patient and family regarding the transition process, the underlying disease and the care of the patient’s health - Healthcare-related education (empowerment in relation to treatment, body and symptom awareness; identification of healthcare and social support networks, and counseling regarding adolescence-related topics) - Fostering the creation of groups of patients and families with the same disease, or promoting participation in support groups for patients with orphan diseases.. |
| **Recommendation 15:**  **The following is recommended regarding the profile of the case manager for the process of transitioning patients with XLH** | This professional (usually a nurse) should have knowledge of the comprehensive approach to patients with XLH. Depending on the characteristics and resources of the institution, other options are a general practitioner or a social worker.^13,26^ |
| **Recommendation 16:**  **The following is recommended regarding the frequency of training for the team in charge of transitioning patients with XLH** | - At the time of creating the transition team^25,27^ - Upon arrival of new members to the team^25,27^ - In case of change of the coordinator or other members of the transition team^25,27^ - Every 12 months for the purpose of adjusting processes in accordance with identified needs^25,27^ - Whenever there is a need for significant changes to the process, the work of the team, or the management, follow-up, and improvement plans^25-27,42^ - Whenever new relevant information emerges, calling for an update^25-27,42^ |
| **Recommendation 17:**  **The following is recommended regarding the frequency with which the content of training for the transition team working with patients with XLH should be updated:** | - The training content should be updated on an annual basis or, alternatively, whenever new relevant medical evidence emerges^25,43^ |
| **Recommendation 18:**  **The following is recommended regarding the frequency of training for patients and families on the transition process in XLH** | - Education programs pertaining to specific aspects of the disease and more general health topics and information on the health system are needed in order to help patients achieve full autonomy. They could be carried out according to the following schedule^25-27,44,45^: - At the time of entering the transition program - Online or face-to-face lectures every 4 to 6 months by age groups or by transition stage - Meetings every 6 months to refresh concepts, reinforce the need for follow-up, and treatment if needed, or to ask specific questions from individual patients - At any time in case the need for additional training or in-depth information on a specific topic is identified at the individual or group level - At each visit with the transition team |
| **Recommendation 19:**  **The following is recommended in order to inform patients with XLH and their families of the activities of the team during the transition process:** | - A formal meeting with the whole team at the time of initiating the transition process to introduce the program and the participating professionals. The transition manager must also be introduced in order to facilitate communication with the family and build trust around the process.^13,25,27,43^ - Follow-up visits for each patient in the transition program in order to update individual activities and to provide continuous training for the patient and the family member.^25,27^ - Different options for conveying information (according to availability and institutional policies): institutional website, brochures, infographics, short update e-mail messages and instant messaging apps, among others.^13,26^ - The frequency varies depending on individual patient needs and characteristics. |
| **Recommendation 20:**  **The following is recommended regarding the person in charge of informing patients with XLH and their families about the activities conducted by the team as part of the transition process:** | This task should be the joint responsibility of the leader of the interdisciplinary transition team (endocrinologist, nephrologist) and the transition manager.^13,25-27^ |
| **Recommendation 21:**  **The following is recommended regarding the timing for informing patients with XLH and their families about the activities conducted by the team as part of the transition process:** | - At the initiation of the transition process, introducing the program and the participating professionals, in order to inform the patient about the minimum requirements associated with the process^13,25,46^ - During the visits scheduled as part of the transition process for each patient, in order to provide updates^16,25^ - Plan a meeting every 12 months with each patient to provide information about their individual process. - The frequency and number of informative and educational meetings can vary depending on individual patient needs and characteristics. It is important to consider the possibility of additional meetings in case there is a need to provide information about new developments, significant changes regarding the treatment, or the transition process itself^13,25^ |
| **Recommendation 22:**  **The following is recommended regarding the assessments required during each follow-up visit in terms of clinical, paraclinical and imaging findings once the pediatric patient with XLH initiates the transition process:** | - Treatment monitoring, clinical and laboratory findings, radiological, dental, neurosurgical, rheumatological, cardiovascular, renal and audiological assessment^5,6,13,31,47^ - Clinical considerations - Thorough clinical history and physical examination: Weight, height, body mass index, blood pressure, growth velocity, pubertal development, presence of pain, lower limb axis and deformities, with intercondylar and intermalleolar distance measurement, and functional capacity - Symptoms, disease complications (headache, dental disease, maxillofacial cellulitis, periodontal disease, musculoskeletal pain, pseudofractures, fatigue, depressive or emotional symptoms) - Treatment adherence, tolerance, efficacy perception and adverse effects   Updated interdisciplinary assessments.   - Paraclinical considerations - Laboratory tests:   - In serum: Phosphorus, calcium, parathormone hormone, alkaline phosphatase, creatinine, 25 OH vitamin D and 1,25 OH vitamin D.   - In urine: Urinalysis, calcium, phosphorus and creatinine (in urine, isolated sample) - Imaging - Based on the individualized assessment, ask for a renal ultrasound and long bone X-rays (general, no specific time interval) |
| **Recommendation 23:**  **The following is recommended regarding the assessment of psychosocial factors during every follow-up visit, once pediatric patients with a diagnosis of XLH initiate the transition process:** | - At each follow-up visit, assess all aspects that can either hinder or ease the transition to adult life, including the degree of overprotection. Additionally, psychosocial and socio-familial questionnaires should be used to gather information regarding family, financial or other issues that might impact the transition process.^13,26,27,46,47^ - Patient emotional and cognitive maturity, self-care capability, autonomy and independence - Knowledge of the disease, laboratory tests and treatment (dose, ability to obtain the medication, what to do in case of adverse reactions or skipping a dose, etc.) - Adherence to treatment - Attendance to appointments (can patients take responsibility for obtaining a follow-up appointment, attending appointments on their own, asking for an appointment in case of changes in their health condition) - Ability to communicate and ask questions of healthcare professionals. - Performance at school and/or work - Self-perception and perception of their own health. Self-esteem. - Functionality of the family and of the support networks. Interactions with peers or orphan disease support groups. - Patient and family opinion regarding the transition process. Level of knowledge regarding the process. - During the visit, questionnaires can be used to determine patient readiness. These include the Transition Readiness Assessment Questionnaire (TRAQ) 5.0, validated in Argentina (includes 20 items in 5 sub-scales: medication, appointment attendance, monitoring of health problems, communication with healthcare professionals and coping with activities of daily living). This is a self-administered tool and each item is scored using a Likert scale from 1 to 5 (1 being minimum autonomy and 5 being maximum autonomy.^25,26^ |
| **Recommendation 24:**  **The following is recommended regarding the maximum interval between follow-up visits for patients with XLH who are in the transition process:** | - This aspect has not been clearly defined in the literature. However, it may vary significantly depending on patient and family profile and needs.^13,25^ Follow-up frequency in patients with XLH must be individualized. Children and adolescents in a rapid growth phase or with changes in medication dose or treatment type should be reassessed every 3 months. Patients with stable clinical and laboratory findings and no therapeutic changes should be reassessed every 6 months. ^13,25^ |
| **Recommendation 25:**  **The following is recommended regarding the minimum number of follow-up visits in patients with XLH who are in the transition process:** | - It depends on the course of the disease and patient and family response to the transition process. ^13,25^ - In the event the medical team in charge of the transition is the main treating team, 3 visits in the year are recommended, depending on the time of initiation of the transition process.^5^ |
| **Recommendation 26:**  **The following is recommended regarding the requirements to determine that the transition process has come to an end in patients with XLH:** | - Acknowledgment of the chronicity of the disease^25,26,35^ - Patient autonomy and empowerment^25,26,35^ - Pharmacological treatment and adjunct therapies not in the process of changing^25,26,35^ - Adherence to treatment^25,26,35^ - Follow-up attendance and a thorough and adequate assessment by the receiving team^25,26,35^ - Patient in stable clinical and metabolic condition^25,26,35^ - Absence of neurological disability or cognitive decline^25,26,35^ |
| **Recommendation 27:**  **The following is recommended regarding the circumstances in which the final visit to close the transition process in patients with XLH should be postponed:** | - Clinical deterioration or development of acute complications^13,25^ - Need to change the treatment^13^ - Non-adherence to the indications and management plan proposed by the receiving team^25^ - Evidence of incomplete or inadequate adaptation to the new treating team by the patient, the family or the transition team^25^ - Family or personal patient dysfunction, with negative impact on adaptation during the transition process^25^ - Absence of patient autonomy, empowerment or acknowledgement of the disease^13,25^ |
| **Recommendation 28:**  **Regarding verification of adequate adaptation to the adult service by patients with X-linked hypophosphatemia who have completed the transition process, the recommendation is to carry out the process in the following way:** | - Telephone call after the first appointment with the adult treating team^25^ - Follow-up by phone according to the characteristics and needs of the patient and family ^48^ - Follow-up visit 6 months to 1 year after transfer to the adult service, including assessment of the following:^25,26^ - Self-care capability and adherence to treatment - Quality of life (anxiety, isolation, self-esteem and self-perception of the health condition) - Patient perspectives regarding the results of the treatment - Questions/satisfaction surveys for the patient and family regarding their adaptation to the process^25^ - Review of the opinion of the receiving treating team regarding patient attitude and adaptation to the process^25^ - Oversight of attendance to appointments and follow-up visits with the receiving team, and adherence to treatment^25^ |
| **Recommendation 29:**  **The following is recommended regarding information about patients with XLH that should be shared among services involved in the transition process:** | Complete information about medical care provided in the pediatric hospital service shared with the adult team that will take over the care of the patient in the future.^13,14,25^   - Patient identification information - Thorough medical history   - - Date of XLH diagnosis     - Description of the disease and comorbidities     - Time on follow-up     - Course of the disease     - Clinical and paraclinical assessment     - Treatments received: Date of initiation and discontinuation, dosing, adverse effects, tolerance, efficacy, adherence.     - Complications associated with the underlying disease or the medications.     - Interdisciplinary assessment reports     - Appointments for interdisciplinary assessments and pending paraclinical tests - Family and social history, and report - Patient schooling level - Risky behaviors - Pattern of coping with the disease and treatment - Level of independence, autonomy and self-care - Any other relevant information that the transition manager or the pediatric treatment group considers important about the patient or relevant for the transition. |
| **Recommendation 30:**  **The following is recommended regarding the indicators that could be considered for reviewing the performance of the team and the impact of the transition process on patients with XLH:** | Quality indicators based on the proposed goals, and objective and reproducible measurements:^13,25,26,49–52^   - Percentage of patients who complete the entire transition process. - Percentage of patients who continue on adequate follow-up with the receiving group for a period of time. - Patient and family perception of the care experience (self-care capability) - Visual analog scale to measure patient and family satisfaction with the transition process - Physician opinion survey regarding medical care transition (use of the transition plan designed with the team, understanding and achievement of the proposed goals, attendance and compliance with the proposed objectives) - Indicator of attendance to scheduled appointments. - Commitment and adherence to pharmacological treatment and management by the receiving team |
| **Recommendation 31:**  **The following is recommended regarding the timing for assessing compliance with the objectives of the transition process for patients with XLH:** | - Regular assessments to determine compliance with objectives by the transition team, the patient and the family during the different stages of the process, documenting achievement of the proposed goals^5,25,26,51^ - Assessments are suggested at the time of the closure visit and 12 months afterwards^25^ |
| **Recommendation 32:**  **The following is recommended regarding documentation of the assessment process to determine compliance with the objectives of the transition in patients with XLH:** | - Design and use of tools and checkli for the transition team, the patient and the family:^13,25,26,53^ - Documenting checklists of proposed and performed activities, and of adequate compliance. - Completion of review and verification lists of key activities at each point in the process and at the end thereof: number of consultation visits, time required to achieve the objectives, follow-up at the end of the transition, and percentage of patients lost to follow-up. - Completion and documentation of the related quality indicators |
| **Recommendation 33:**  **The following is recommended regarding the minimal requirements that have to be met by the health system in order to ensure the development of an adequate transition process in patients with XLH:** | - Ensure:^13,25,26,53^ - Complete funding for the transition clinical unit in order to allow its comprehensive development. - Specialists who participate in the transition process in the same institution or connected in such a way as to allow patient care to flow seamlessly. - Adoption of updated scientific recommendations regarding the care of patients with XLH - Availability of physical facilities to conduct the process, including meetings, planning, execution, and joint pediatric and adult consultations. - Adequate availability and coverage to allow easy access to the health system for patients, diagnostic tests, interdisciplinary assessments and comprehensive treatment. |
